# Incidence of SARS-CoV-2 infection in children shortly after ending zero-COVID-19 policy in China on December 7, 2022: a cross-sectional, multicenter, seroepidemiological study

**DOI:** 10.3389/fpubh.2023.1283158

**Published:** 2023-11-09

**Authors:** Yi-Hua Zhou, Chenyu Xu, Yue Tao, Meng Gu, Guiping Zhou, Wei Zhou, Yue Jin, Jun Xie, Biyun Xu, Wensan Zhou, Junhao Chen, Weifeng Shi

**Affiliations:** ^1^Departments of Laboratory Medicine and Infectious Diseases, Nanjing Drum Tower Hospital, Affiliated Hospital of Medical School, and Jiangsu Key Laboratory for Molecular Medicine, Nanjing University, Nanjing, Jiangsu, China; ^2^Department of Obstetrics and Gynecology, Zhenjiang Fourth People’s Hospital, Zhenjiang, Jiangsu, China; ^3^Department of Clinical Laboratory, Affiliated Changzhou Children's Hospital of Nantong University, Changzhou Children's Hospital, Changzhou, Jiangsu, China; ^4^Department of Clinical Laboratory, Yixing Second People’s Hospital, Yixing, Jiangsu, China; ^5^Department of Clinical Laboratory, The First People’s Hospital of Changzhou, Changzhou, Jiangsu, China; ^6^Department of Clinical Laboratory, Huai'an Second People's Hospital and the Affiliated Huai'an Hospital of Xuzhou Medical University, Huai’an, Jiangsu, China; ^7^Department of Clinical Laboratory, People's Hospital of Xuyi County, Xuyi, Jiangsu, China; ^8^Medical Statistics and Analysis Center, Nanjing Drum Tower Hospital, Affiliated Hospital of Medical School, Nanjing University, Nanjing, Jiangsu, China

**Keywords:** antibody to SARS-CoV-19, prevalence, children, COVID-19 vaccination, discontinuation of zero-COVID-19 policy

## Abstract

**Background:**

China discontinued the zero-COVID-19 policy on December 7, 2022, and then COVID-19 surged mid-December 2022 through mid-January 2023. However, the actual incidence was unknown. This study aimed to estimate the incidence of SARS-CoV-2 infection in children shortly after ending the zero-COVID-19 policy.

**Methods:**

This multicenter cross-sectional study included 1,065 children aged 8 months to 12 years from seven hospitals at six regions across Jiangsu province, based on the convenience sampling, from February 10 to March 10, 2023. Group I comprised 324 children aged 8 months–2 years without COVID-19 vaccination, group II consisted of 338 preschool children aged 3–5 years with varied vaccination history, and group III contained 403 primary school children aged 6–12 years with mostly vaccinated. The COVID-19 vaccines were composed of inactivated SARS-CoV-2. In addition, 96 children’s sera collected in 2014 were included as negative controls. IgG and IgM antibodies against nucleocapsid (N) and subunit 1 of spike (S1) of SARS-CoV-2 (anti-N/S1) were measured with commercial kits (YHLO Biotech, Shenzhen, China).

**Results:**

None of the 96 children (5.1 ± 3.5 years; 58.3% boys) in 2014 was positive for anti-N/S1 IgG or IgM. Of the 1,065 children (5.0 ± 3.5 years; 56.0% boys), 988 (92.8%) were anti-N/S1 IgG positive but none was anti-N/S1 IgM positive. The positive rate of anti-N/S1 IgG in Group I, II, and III was 90.4, 88.5, and 98.3%, respectively, with significantly higher in group III than in groups I and II (*p* < 0.0001). The median antibody titers in group III (381.61 AU/ml) were much higher than that in group I (38.34 AU/ml) and II (51.88 AU/ml; *p* < 0.0001).

**Conclusion:**

More than 90% children experienced SARS-CoV-2 infection shortly after ending zero-COVID-19 policy in China, much higher than estimated infections by other studies. The widespread SARS-CoV-2 infection in unvaccinated children should be influential on the policy of COVID-19 vaccination in children in the future.

## Introduction

Severe acute respiratory syndrome coronavirus 2 (SARS-CoV-2), the etiology of coronavirus disease 2019 (COVID-19), is transmitted through respiratory tract by inhaling the virus in droplets and aerosol. China had taken the extremely strict non-pharmaceutical interventions to contain SARS-CoV-2, called “zero-COVID-19 policy,” after recognition of the disastrous consequences of COVID-19 in early 2020 (1, 2). The zero-COVID-19 policy played important roles in the control of COVID-19 in the mainland China. After the control of the large outbreak of COVID-19 initially occurred in Wuhan city and complete block of transmission of SARS-CoV-2 throughout mainland China in early April, 2020, only transient sporadic clusters of COVID-19 cases occurred in different cities or rural areas, which were completely controlled within 1 to 2 months after the implementation of strict non-pharmaceutical interventions. China has discontinued the strict non-pharmaceutical interventions since December 7, 2022 (3). Shortly thereafter, COVID-19 surged in mainland of China, and the pandemic lasted about 1 month, from middle of December 2022 to middle of January 2023. However, the actual incidence rate of SARS-CoV-2 infection was unknown.

Since December 2020, COVID-19 vaccines have been used in mainland China, initially among healthcare workers (4, 5), and then extended to adult, adolescent, and children populations (6). However, children aged lower than 3 years are not eligible for COVID-19 vaccination, and these children were not immunized with COVID-19 vaccines. Thus, measurement of antibodies to SARS-CoV-2 in them may determine whether they had been infected. In the present study, we tested the IgG and IgM antibodies to SARS-CoV-2 in children to estimate the infection rate of SARS-CoV-2 in children from mid-December 2022 to mid-January 2023, the pandemic period shortly after the discontinuation of zero-COVID-19 policy in China.

## Subjects and methods

### Design and participants

This was a cross-sectional study in collaboration with seven hospitals from six regions across Jiangsu province, which is located in east part of China and has 85 million residents. These hospitals are located in the southern and northern parts of Jiangsu. During February 10–March 10, 2023, we collected 1,065 serum or plasma samples from 1,065 children aged 8 months to 12 years at the collaborating hospitals. Accompanied by parents or guardians, these children visited hospitals for various reasons and required necessary blood tests by venipuncture. After the necessary laboratory tests such as blood routine tests and clinical biochemistry, residual serum or plasma samples were aliquoted and stored at −30°C, which were transported in iceboxes to Nanjing Drum Tower Hospital for subsequent use in the present survey.

In China, COVID-19 vaccination is recommended for children aged ≥3 years and children under the age of 3 years are not vaccinated against COVID-19. In practice, vast majority of primary school children (≥6 years age) were vaccinated because of the requirements of the schools, children aged lower than 3 years were not vaccinated, and the vaccination among children aged 3 to 5 years was determined by parents or guardians. The COVID-19 vaccines used in children were composed of inactivated SARS-CoV-2. We planned to divide children into three groups based on the vaccination status. Based on the estimated anti-SARS-CoV-2 positive rate of 60%, the participant size in this survey was 257 in each group, with a confidence of 95% and a relative error of 10%. We finally included 1,065 children in total, who were divided into three groups. Group I comprised 324 children aged 8 months−2 years who were not vaccinated against COVID-19, group II, 338 children aged 3–5 years who were preschool children with varied vaccination history, and group III, 403 children aged 6–12 years who were primary school students with mostly vaccinated.

In addition, we included 96 children’s serum samples collected in 2014, before the occurrence of COVID-19, to serve as negative controls. The samples were tested for the presence of hepatitis B serological markers in a previous study (7), and were kept at −30°C.

This study was approved by the institutional review board (IRB) of Nanjing Drum Tower Hospital (No. 2023-100-01) and other participating hospitals. Consent from parents/guardians of children was exempted since investigators did not directly contact the children and their parents or guardians and the samples used in this study were residual serum or plasma samples after the necessary laboratory tests.

### Detection of anti-SARS-CoV-2 IgG and IgM antibody

IgG and IgM antibodies to SARS-CoV-2 were measured with chemo-luminescence immunoassay kits (iFlash 3,000 chemiluminescence immunoassay analyzer, Shenzhen YHLO Biotech, China) as described elsewhere (8, 9). The detection antigens in the kits are recombinant nucleocapsid (N) and spike protein S1 subunit (S1) of SARS-CoV-2 synthesized in baculovirus. Thus, the antibodies tested are directed against the N and S1 of SARS-CoV-2 (anti-N/S1). Both anti-N/S1 IgM and IgG assays are widely used worldwide and served as a confirmatory test (10–13). Based on the manufacturer’s instructions, a value equal to or higher than 10.0 arbitrary units (AU)/ml was considered positive, and that below 10.0 AU/ml was considered negative. All samples were measured in Nanjing Drum Tower Hospital.

### Statistical analysis

Continuous variables normally distributed were expressed as mean ± standard deviation (SD), and independent t-test was used to compare differences between groups. Non-normal continuous variables were presented as median (minimum–maximum), and the Kruskal-Wallis rank-sum test was used for comparisons of level of anti-N/S1 IgG among three age groups followed by Nemenyi post-hoc test for multiple comparisons. The 95% confidence intervals (CI) were reported for positive rates of anti-N/S1 IgG. Categorical data were presented as percentages, and χ^2^ tests were performed to analyze the differences between groups with Bonferroni’s multiple comparison. A two-sided *p* value of <0.05 was considered as statistically significant. All statistical analyses were conducted using the SPSS 25.0 (version 25.0, SPSS, Chicago, IL, USA).

## Results

None of the serum samples collected in 2014 from 96 children (mean age, 5.1 ± 3.5 years; 56 [58.3%] boys) was positive for anti-N/S1 IgM or anti-N/S1 IgG. Of the 1,065 children enrolled February 10 to March 10, 2023, 596 (56.0%) were boys, and the mean age was 5.0 ± 3.5 years, with 5.0 ± 3.5 in boys and 5.1 ± 3.4 in girls (t = 0.4687, *p* = 0.6394). None (0%) of them was anti-N/S1 IgM positive, and 988 (92.8%) were anti-N/S1 IgG positive and 77 (7.2%) were anti-N/S1 IgG negative. The positive rate (91.4%, 545/596) of anti-N/S1 IgG in boys was comparable to that (94.5%, 443/469) in girls (*χ*^2^ = 3.553, *p* = 0.059; Table 1).

**Table 1:** Positive rate of anti-N/S1 IgG in children at different ages.

Age group	No (%)^*^	Age, years	Positive No.	Positive rate, %^†^ (95% CI)
All ages	1,065	5.0 ± 3.5	988	92.8 (91.2–94.3)
Boy	596 (56.0)	5.0 ± 3.5	545	91.4 (89.2–93.7)^§^
Girl	469 (44.0)	5.1 ± 3.4	443	94.5 (92.4–96.5)^§^
I: 8 months–2 years	324	1.4 ± 0.5	293	90.4 (87.2–93.7)
Boy	186 (57.4)	1.4 ± 0.5	169	90.9 (86.7–95.0)
Girl	138 (42.6)	1.4 ± 0.6	124	89.9 (84.8–95.0)
II: 3–5 years	338	3.8 ± 0.8	299	88.5 (85.0–91.9)
Boy	189 (55.9)	3.8 ± 0.8	161	85.2 (80.1–90.3)
Girl	149 (44.1)	3.9 ± 0.9	138	92.6 (88.4–96.9)
III: 6–12 years	403	9.0 ± 2.1	396	98.3 (97.0–99.5)
Boy	221 (54.8)	9.1 ± 2.1	215	97.3 (95.1–99.4)
Girl	182 (45.2)	8.8 ± 2.1	181	99.5 (97.0–99.99)

* The difference in the proportion of boys among the three groups was not statistically significant (*χ*^2^ = 0.4813, *p* = 0.7861).

† The difference in positive rate of anti-N/S1 IgG among three groups was statistically significant (*χ*^2^ = 30.1239, *p* < 0.0001). The positive rate in group III was significantly higher than that in children in group I and II (*χ*^2^ = 22.2335, *p* < 0.0001; *χ*^2^ = 30.3306, *p* < 0.0001), whereas the difference between group I and II was not statistically significant (*χ*^2^ = 0.679, *p* = 0.410).

§ The positive rate between boys and girls had no statistical difference (*χ*^2^ = 3.553, *p* = 0.059).

**Figure 1:**
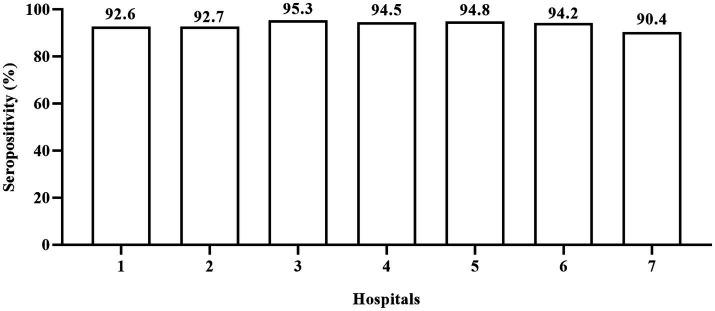
Positive rate of IgG antibody to nucleocapsid and subunit 1 of spike proteins of SARS-CoV-2 in children at different hospitals. 1–6: represent general hospitals; 7, Children’s hospital. The anti-N/S1 IgG positive rates had no statistical difference (*χ*^2^ = 6.234, *p* = 0.397) in children from these hospitals.

Since the children included in this study were from seven hospitals from six regions of Jiangsu province, we compared the positive rate of anti-N/S1 IgG in children from the different hospitals. Figure 1 shows the anti-N/S1 IgG positive rates in children from these hospitals, ranging from 90.4 to 95.3%, and the difference had no statistical significance (*χ*^2^ = 6.234, *p* = 0.397).

As the children aged 8 months–2 years were not vaccinated, most of children aged 6–12 years were vaccinated and the children aged 3–5 years had varied vaccination history, we further compared the positive rates of anti-N/S1 IgG among these three groups. The proportion of boys in each group was 57.4, 55.9, and 54.4%, respectively, and the difference had no statistical significance (Table 1). The positive rate of anti-N/S1 IgG in each group is shown in Table 1, with 90.4% in group I, 88.5% in group II, 98.3% in group III. The positive rate in group III (children aged 6–12 years) was significantly higher than those in children in group I and II, while the positive rates had no statistical significance between group I (8 months–2 years) and II (3–5 years).

**Figure 2:**
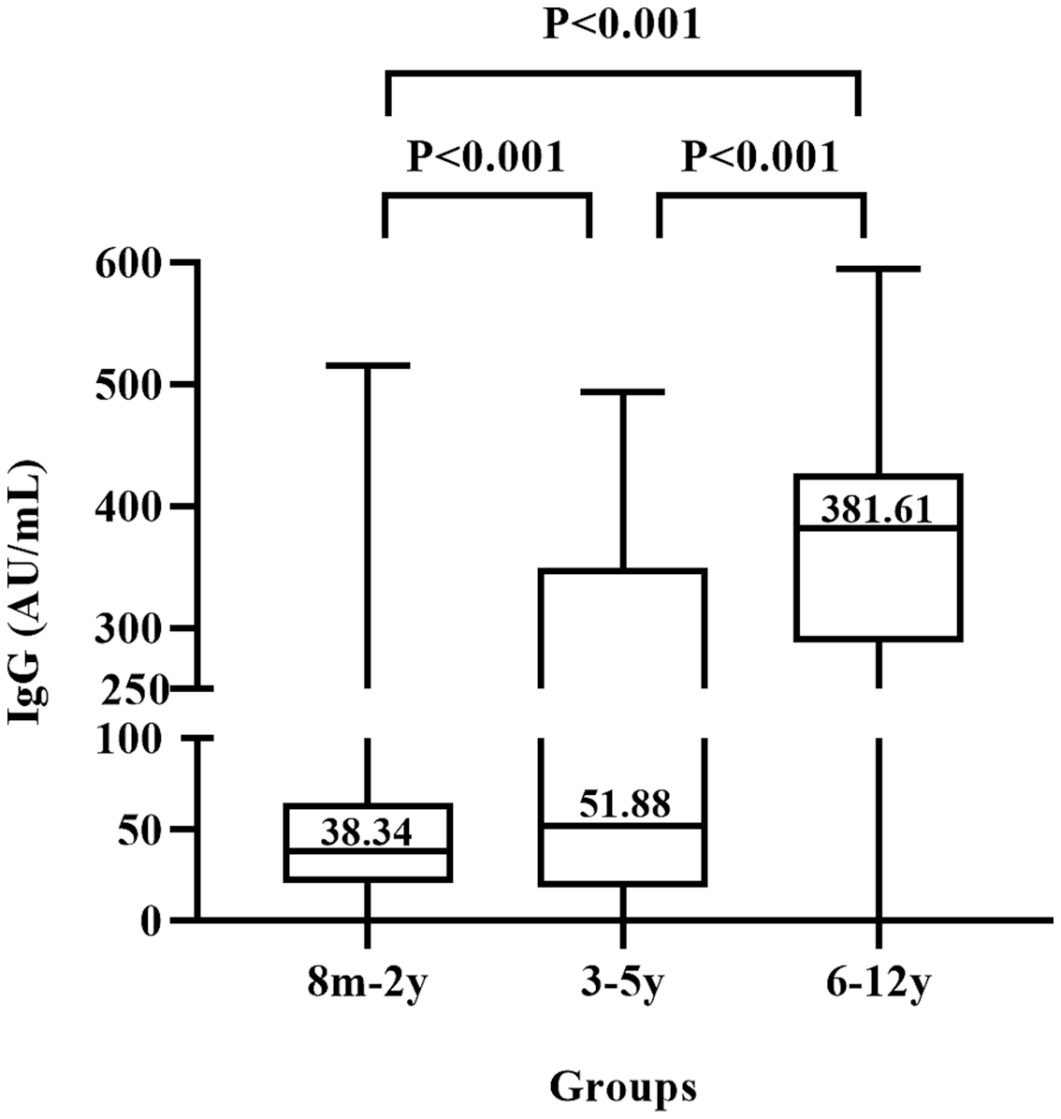
Titers of IgG antibody to nucleocapsid and subunit 1 of spike proteins of SARS-CoV-2 in children. 8 m–2 y represents 324 children aged 8 months to 2 years who were not vaccinated against COVID-19, 3–5 y represents 338 children aged 3–5 years who had varied vaccination history, and 6–12 y represents 403 children aged 6–12 years who were mostly vaccinated.

Since the children in the three groups had different COVID-19 vaccination history, we compared the median titers of anti-N/S1 among these children (Figure 2). Antibody titers among the three groups had statistically significant difference (*χ*^2^ = 313.6381, *p* < 0.0001), and further comparison showed that antibody titers (381 AU/ml) in group III (6–12 years old children) were significantly higher than that in group I (8 months–2 years, 38.34 AU/ml) and group II (3–5 years, 51.88 AU/ml) and antibody titers in group II children were also significantly higher than that in group I (*p* < 0.001).

## Discussion

In the present survey, we revealed that the positive rate of anti-N/S1 IgG in children aged 8 months to 12 years in Jiangsu was 92.8%, and even in young children who were not vaccinated against COVID-19, the anti-N/S1 IgG positive rate was as high as 90.4%. Based on the daily epidemiologic data in mainland China disclosed by the central, provincial, and local health authorities, there were only a few children who were defined with COVID-19 in Jiangsu before December 7, 2022, which is negligible in the calculation of COVID-19 incidence after ending the zero-COVID-19 policy. Therefore, our results suggest that more than 90% of the children in Jiangsu province were infected with SARS-CoV-2 during mid-December 2022 to mid-January 2023, shortly after the ending of the zero-COVID policy in December 7, 2022.

The 324 children aged 8 months to 2 years included in the present study were not vaccinated with COVID-19 vaccines because of the COVID-19 vaccination policy in China. Although maternal IgG antibodies can transplacentally transfer into fetus, maternally derived anti-SARS-CoV-2 IgG usually disappears in infants by the 6 months age. In addition, the mothers of these children were not infected with SARS-CoV-2 during their pregnancy, and intrauterine transmission of SARS-CoV-2 is very rare (10, 11). These children were unlikely to have anti-SARS-CoV-2 due to the intrauterine infection. Therefore, the finding of anti-N/S1 IgG positive in 90.4% of children aged 8 months to 2 years in the present study demonstrates that they experienced SARS-CoV-2 infection.

Because of the requirement of stringent zero-COVID-19 policy in China, most primary school students (at the age of 6–12 years) were vaccinated against COVID-19. In the present survey, 98.3% of these children were anti-SRAS-CoV-2 IgG positive (Table 1). One may assume that the antibodies in these children were elicited by the vaccination. However, those children with a few months old than 6 years in the sample collection period (February 10 to March 10, 2023) were less likely vaccinated, since China school year starts September 1 of each year and they were less than 6 years old in September of 2022. Moreover, the significantly higher (around 10-fold) titers of anti-N/S1 IgG (Figure 2) suggest a booster caused by natural infection, because vaccination with inactivated COVID-19 vaccines usually induces relatively low antibody titers and wanes rapidly within several months (12).

While China recommends vaccinate children aged ≥3 years, preschool age children (3–5 years old) had considerably variable vaccination coverage because whether vaccination in these children was determined by parents or guardians. In the present study, 88.5% of these children were anti-N/S1 IgG positive. Since the detailed vaccination information in these children was not available, whether the antibodies were resultant from vaccination or from natural infection remains cannot be definitely determined. However, we consider that it was likely that these children underwent natural infection because of the following reasons. First, those children with a few months old than 3 years in the sample collection period (February 10 to March 10, 2023) were unlikely vaccinated, since they were less than 3 years old in December of 2022. Second, the COVID-19 vaccination coverage in these children was lower based on the previous investigations (13). Third, the antibody titers in this child group were much lower than that in children aged 6–12 years and slightly higher than that in the 8 months–2 years group (Figure 2), suggesting that they were mostly not vaccinated.

Noticeably, none of the 1,065 children, including 988 children with positive anti-N/S1 IgG, was positive for anti-N/S1 IgM. This is unlikely associated with the performance of the assay, because this reagent is widely used worldwide (14–16) and anti-N/S1 IgM was easily detected by the agents (17, 18). Studies demonstrated that anti-N/S1 IgM responses in symptomatic COVID-19 patients are transient and largely fall below the detection limit 30 days after symptom onset (19, 20). The absence of anti-N/S1 IgM indicates that the infection in children occurred shortly after the ending of the zero-COVID-19 policy in China in December 7, 2022, as the children’s blood samples were collected from February 10 to March 10, more than 2 months after the ending of the zero-COVID-19 policy.

Interestingly, children aged 6–12 years had very significantly higher (10-fold) antibody titers than children aged <3 years (Figure 2), which is less likely associated with the different ages, since young children can also mounted robust antibody response after natural infection (21). The big difference in antibody titers may be related to the different status of vaccination history, since children aged <3 years were not vaccinated and most of children aged 6–12 years were likely vaccinated. Thus, vaccinated children, if breakthrough infection occurred, may produce much more potent antibody response, which will be useful to protect against COVID-19 for a longer duration if exposed again to SARS-CoV-2 in the future.

The present survey provides important information about the incidence rate of SARS-CoV-2 infection shortly after the ending of zero-COVID-19 policy in China. Children aged 8 months to 2 years had the least chance to expose to SARS-CoV-2 because the daycare centers were almost all closed then and they were taken cared at home. They should have the least possibility to be infected; however, 90.4% of them were infected. Moreover, none of them was IgM positive, indicating that the infection occurred at least 1–2 months ago. As the blood samples were collected February 10 to March 10, 2023, it is plausible to consider that vast majority, if not all, of infections occurred from mid-December 2022 to mid-January 2023. Our findings are roughly consistent with the findings in Guangzhou city (south part of China) that the incidence rate of COVID-19 in 1500 patients aged 1–99 years during January 5 to 14, 2023 was 80.7% (22). Moreover, we found that children from seven hospitals at six cities and rural areas had similar positive rates of anti-SARS-CoV-2 (Figure 1), indicating that the results are less likely to be biased because of the convenience sampling. Thus, our finding that over 90% of children experienced COVID-19 during 1 month period in the present study may suggest that a similar proportion of children in other parts of mainland China were probably infected during the same period. Thus, the actual COVID-19 incidence rate during mid-December 2022 to mid-January 2023 may be much higher than the incidence rate estimated by positive results of SARS-CoV-2 RNA or antigens (23). The estimated infection rate of SARS-CoV-2 in our study is also much higher than the estimated infection number in Beijing, weeks 2–6 of 2023, based on the influenza surveillance system, in which the overall incidence of SARS-CoV-2 infection was only 0.392% during the 5 weeks following ending the zero-COVID-19 policy (24).

The high infection rate in the children aged 8 months to 2 years (not eligible for COVID-19 vaccination) raised an issue of whether these children should receive COVID-19 vaccination when they become eligible for vaccination over time. In addition, the duration of antibodies directed against SARS-CoV-2 in these young children is unknown, which is also important for the determination of vaccination against COVID-19. The very significantly higher anti-SARS-CoV-2 titers in vaccinated and infected children (Figure 2) suggest that the protection duration may be much longer. These issues merit further study.

There are some limitations in our present study. First, the children were selected from those who sought medical examinations or treatments based on convenience sampling, but not selected based on the whole child population in Jiangsu province. To overcome the potential bias, we enrolled the children from seven hospitals located at six regions across the Jiangsu Province, which may roughly represent the child population at the age of 8 months–12 years. Second, while children aged 8 months–2 years were surely not vaccinated, the vaccination information in children aged 3–6 years and aged 7–12 years was not validated by checking the vaccination card.

## Conclusion

Our results show that more than 90% of children in Jiangsu China experienced SARS-CoV-2 infection during mid-December 2022 through mid-January 2023 after discontinuation of the zero-COVID-19 policy, much higher than the previously estimated incidence rate of SARS-CoV-2 infection. The impact of this extremely large epidemic on the reemergence of the COVID-19 pandemic in the future merits further observation.

## Data availability statement

The raw data supporting the conclusions of this article will be made available by the authors, without undue reservation.

## Ethics statement

The studies involving humans were approved by the institutional review board (IRB) of Nanjing Drum Tower Hospital (No. 2023-100-01). The studies were conducted in accordance with the local legislation and institutional requirements. The ethics committee/institutional review board waived the requirement of written informed consent for participation from the participants or the participants’ legal guardians/next of kin since investigators did not directly contact the children and their parents or guardians and the samples used in this study were residual serum or plasma samples after the necessary laboratory tests.

## Author contributions

Y-HZ: Conceptualization, Funding acquisition, Supervision, Validation, Writing – original draft. CX: Conceptualization, Resources, Supervision, Validation, Writing – original draft. YT: Investigation, Methodology, Resources, Validation, Writing – original draft. MG: Investigation, Methodology, Resources, Validation, Writing – review & editing. GZ: Investigation, Methodology, Resources, Writing – review & editing. WeiZ: Investigation, Methodology, Validation, Writing – review & editing. YJ: Investigation, Methodology, Resources, Validation, Writing – review & editing. JX: Investigation, Methodology, Resources, Writing – review & editing. BX: Formal analysis, Methodology, Software, Writing – original draft. WenZ: Investigation, Resources, Supervision, Validation, Writing – review & editing. JC: Conceptualization, Methodology, Project administration, Resources, Writing – review & editing. WS: Conceptualization, Project administration, Resources, Supervision, Visualization, Writing – review & editing.
